# Two-Year Implementation, Adherence, and Outcomes of Quadruple Guideline-Directed Medical Therapy in Newly Diagnosed HFrEF: Insights from the Prospective CaRD Registry

**DOI:** 10.3390/jcm15062127

**Published:** 2026-03-11

**Authors:** Ivana Jurin, Daniel Lovrić, Karlo Gjuras, Šime Manola, Irzal Hadžibegović, Mario Udovičić, Diana Rudan, Anica Milinković, Jasmina Ćatić, Marija Križanović, Marin Pavlov

**Affiliations:** 1 Department of Cardiovascular Diseases, University Hospital Dubrava, 10000 Zagreb, Croatia; sime.manola@icloud.com (Š.M.); irzalh@gmail.com (I.H.); mario.udovicic@gmail.com (M.U.); drudan3@yahoo.com (D.R.); jcjasmina@gmail.com (J.Ć.); marin.pavlov@gmail.com (M.P.); 2 Department of Cardiovascular Diseases, University Hospital Centre Zagreb, 10000 Zagreb, Croatia; daniel@dlovric.net (D.L.); anica.milinkovic18@gmail.com (A.M.); 3 Department of Family Medicine, Health Centre Bjelovar-Bilogora County, 43000 Bjelovar, Croatia; karlogjuras4@gmail.com; 4 School of Medicine, University of Zagreb, 10000 Zagreb, Croatia; marija.krizanovic1510@gmail.com; 5 Faculty of Dental Medicine and Health Care, Josip Juraj Strossmayer University of Osijek, 31000 Osijek, Croatia; 6 Department of Nursing, University North, 48000 Koprivnica, Croatia

**Keywords:** heart failure, reduced ejection fraction, guideline-directed medical therapy, adherence, titration, real-world data

## Abstract

**Background**: Contemporary guidelines recommend rapid initiation of four classes of guideline-directed medical therapy (GDMT) for heart failure (HF) with reduced ejection fraction (HFrEF); however, real-world persistence, adherence, and dose optimization remain suboptimal. **Methods**: We analysed a predefined subregistry within the prospective Cardiology Research Dubrava (CaRD) registry, a real-world HF registry at a tertiary centre that includes patients across the ejection-fraction spectrum in whom contemporary HF therapy, including sodium-glucose cotransporter 2 inhibitors (SGLT2i), is introduced or optimised in routine practice. For this analysis, we included patients with newly diagnosed HFrEF (left ventricular ejection fraction (LVEF) ≤ 40%) who were discharged on all four GDMT classes; 167 of 179 patients with newly diagnosed HFrEF during the study period had an available 6-month medication assessment and comprised the final analytic cohort. The four GDMT pillars (beta-blocker; angiotensin-converting enzyme inhibitor (ACEi), angiotensin receptor blocker (ARB), or angiotensin receptor-neprilysin inhibitor (ARNI); mineralocorticoid receptor antagonist (MRA); and SGLT2i) were initiated within 4 days when clinically feasible. Medication adherence and target-dose attainment were assessed at 6, 12, and 24 months using a structured self-report questionnaire. Major adverse events (MAE) and all-cause mortality were recorded over 24 months. Patients were classified as adherent if they reported regular intake (≥80% of prescribed doses) of all four drug classes at 6 months; otherwise, they were classified as nonadherent. **Results**: Among the 167 analysed patients (median age 64 years, 74% men, median LVEF 30%), regular adherence at 6, 12, and 24 months was 65%, 55%, and 59% for beta-blockers; 66%, 50%, and 49% for ACEi/ARB/ARNI; 62%, 52%, and 49% for MRAs; and 84%, 57%, and 68% for SGLT2i. Target doses were achieved in 25–33% for beta-blockers, 42–50% for ACEi/ARB/ARNI, and 73–78% for MRAs. At 24 months, 56 survivors (37%) were adherent to all four drug classes. Over 24 months, all-cause mortality was 9.0% and MAE 18.6%, occurring less frequently in adherent vs. nonadherent patients (mortality 0% vs. 13.5%; MAE 8.9% vs. 23.4%). **Conclusions**: In this real-world, non-randomized HFrEF subregistry, in-hospital initiation of quadruple GDMT was feasible, yet maintaining long-term adherence and achieving target doses remained challenging. These data underscore the gap between guideline recommendations and routine practice and support structured follow-up and protocol-driven titration to optimize implementation.

## 1. Introduction

Contemporary heart failure (HF) guidelines recommend early initiation of four foundational drug classes—renin–angiotensin system inhibition with an angiotensin-converting enzyme inhibitor (ACEi), angiotensin receptor blocker (ARB) or angiotensin receptor–neprilysin inhibitor (ARNI), an evidence-based beta-blocker (BB), a mineralocorticoid receptor antagonist (MRA), and a sodium–glucose cotransporter-2 inhibitor (SGLT2i)—for patients with heart failure with reduced ejection fraction (HFrEF), based on robust evidence demonstrating rapid and clinically meaningful reductions in morbidity and mortality [1,2]. Each of these therapies has been shown to confer early prognostic benefit, supporting strategies of rapid or near-simultaneous initiation rather than traditional stepwise escalation [3,4,5,6,7,8,9,10,11,12,13,14].

Despite strong guideline endorsement, real-world implementation of quadruple guideline-directed medical therapy (GDMT) remains challenging. Large registries consistently demonstrate substantial gaps in prescription rates, long-term persistence, and achievement of guideline-recommended target doses, particularly beyond the early post-discharge period [15,16,17,18,19]. Recent implementation frameworks have therefore emphasized that the clinical benefit of GDMT depends not only on early initiation but also on sustained use and appropriate dose optimisation over time [11,12,13,14]. High-intensity, protocol-driven follow-up strategies have been proposed to address these gaps. In the STRONG-HF trial, rapid initiation and aggressive up-titration of guideline-recommended therapies combined with close post-discharge follow-up significantly reduced 180-day heart failure readmissions and all-cause mortality compared with usual care [20]. However, even in this optimized setting, data on long-term persistence, drug-class–specific adherence patterns, and patient-reported barriers beyond the early post-discharge period remain limited.

Against this background, we evaluated initiation, persistence and dose optimization of the four foundational therapies in a prospective cohort of patients with newly diagnosed HFrEF. Specifically, we aimed to: (1) describe uptake of quadruple therapy at discharge; (2) quantify adherence and target-dose achievement for each drug class at 6, 12 and 24 months; (3) identify patient and socioeconomic correlates of nonadherence; and (4) explore associations between adherence to all four classes and 2-year clinical outcomes.

## 2. Materials and Methods

This prospective observational cohort study analysed a predefined subregistry of the Cardiology Research Dubrava (CaRD) registry (ClinicalTrials.gov identifier NCT06090591), a broader prospective HF registry at our tertiary centre that includes patients across the ejection-fraction spectrum (preserved, mildly reduced, and reduced) in whom contemporary HF therapy, including SGLT2i, is initiated or optimised in routine clinical care, between April 2022 and December 2023 (Supplementary Material). For the present analysis we focused on patients with newly diagnosed HFrEF who were discharged on all four foundational GDMT classes and followed them in greater detail to assess how feasible sustained quadruple therapy is in non-randomized patients encountered in everyday clinical practice. Patients were considered newly diagnosed if HFrEF (left ventricular ejection fraction (LVEF) ≤ 40%) was identified for the first time during the index hospitalisation. In this manuscript, the term “2-year” refers to the intended patient-level 24-month follow-up after the index date rather than to a fixed registry calendar cut-off. The analysis was based on routinely collected registry data and was approved by the Institutional Ethics Committee. All patients provided informed consent for the use of anonymized data for research and publication purposes. The study was conducted in accordance with the Declaration of Helsinki.

**Figure A1:**
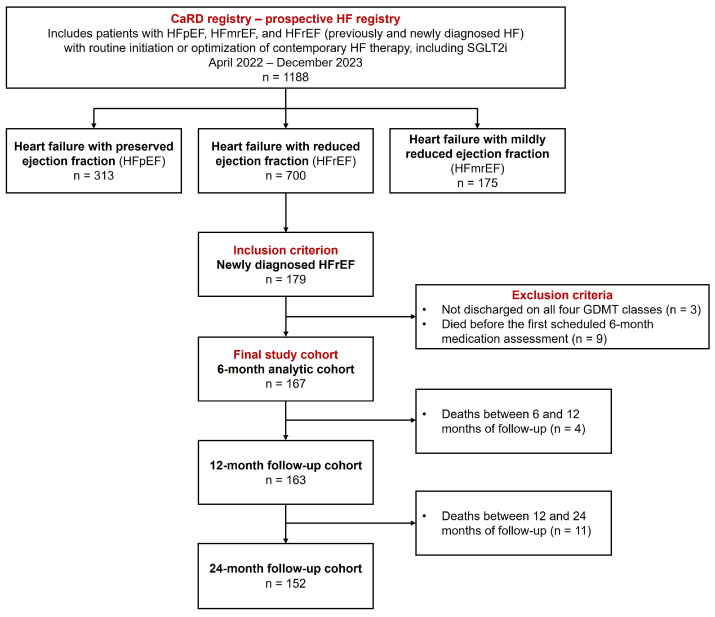
Study flowchart of patient selection from the CaRD registry.

For the present analysis, we included only patients with newly diagnosed HFrEF who were prescribed all four foundational GDMT classes (ACEi/ARB/ARNI, BB, MRA and SGLT2i) at hospital discharge and had an available 6-month medication assessment. Accordingly, this analytic cohort was defined by the treating physician’s judgement that initiation of all four pillars was clinically feasible at discharge, rather than by the complete absence of low blood pressure, renal dysfunction, or other potential barriers. Within the broader CaRD HF registry, 179 patients had newly diagnosed HFrEF during the study period; 3 were not discharged on all four drug classes (e.g., due to contraindications, intolerance, or other clinical reasons), and 9 died before the first scheduled 6-month medication assessment, leaving 167 patients in the final analytic cohort (Figure A1).

In eligible patients, the four GDMT pillars were initiated during hospitalization, with a goal to start all four classes within 4 days from diagnosis. Dose titration was performed according to guideline-recommended target doses and individual tolerability. Device therapy (implantable cardioverter-defibrillator and/or cardiac resynchronization therapy) was implemented according to European Society of Cardiology (ESC) guideline indications [1].

For the exploratory adherence-outcome analysis, patients were classified as adherent if they reported regular intake (≥80% of prescribed doses) of all four GDMT pillars at the 6-month assessment. Patients who were irregular or not taking at least one GDMT class were classified as nonadherent. Because adherence status was defined at 6 months, the corresponding outcome analyses should be interpreted as exploratory. We did not use a landmark analysis to describe adherence over time; the 6-, 12-, and 24-month adherence results reflect prespecified longitudinal patient-level assessments, whereas the 6-month classification was used only for the exploratory outcome comparison.

Patients were enrolled during the predefined inclusion period, and follow-up was conducted at 6, 12, and 24 months after the index date, defined as prescription/initiation of quadruple GDMT at hospital discharge. Accordingly, each patient had an individual 24-month follow-up period, irrespective of the calendar year of enrolment. At each visit, a structured medication questionnaire was administered to assess adherence to each GDMT class. This assessment was based on patient self-report and did not include pharmacy refill data, pill counts, or electronic monitoring; the questionnaire was not formally validated against these external adherence measures. In addition, the electronic medical record was reviewed to verify whether patients subsequently attended their family/general practitioner for the prescribed post-discharge therapy, providing supportive information on activation or continuation of treatment in primary care, although this still could not confirm actual medication ingestion. Patients were asked whether they were taking each medication regularly (≥80% of prescribed doses), irregularly (50–80% of prescribed doses) or not taking/rarely taking the medication (≤50% of prescribed doses). For ACEi/ARB/ARNI, BBs and MRAs, target-dose achievement was recorded for patients taking the therapy regularly or irregularly. Reasons for irregular use or discontinuation, as well as reasons for failure to reach target dose, were documented using predefined categories, including poor tolerability/adverse effects, forgetfulness/non-intentional nonadherence, low perceived necessity of treatment, lack of instruction to take the medication, bradycardia, hypotension, renal dysfunction/hyperkalaemia, and lack of attempted up-titration, depending on drug class.

The primary outcomes were all-cause mortality and major adverse events (MAE) over 24 months. MAE included HF decompensation requiring hospitalization, acute coronary syndrome, cerebrovascular events (stroke or transient ischemic attack), thromboembolic or peripheral vascular events, sepsis, dialysis or worsening renal function, and heart transplantation.

### Statistical Analysis

Categorical variables are presented as counts and percentages and were compared using the χ^2^ test. Continuous variables are expressed as mean ± standard deviation or median with interquartile range (IQR), according to data distribution. The normality of continuous variables was assessed using the Kolmogorov–Smirnov test. Between-group comparisons were performed using the Student’s *t* test for normally distributed variables and the Mann–Whitney U test for non-normally distributed variables.

Time-to-event analyses for all-cause mortality and MAE were conducted using Kaplan–Meier estimates and compared with the log-rank test. Survival and MAE were analyzed according to adherence status, comparing adherent versus non-adherent patients separately for each of the four pillars of guideline-directed medical therapy for HFrEF, as well as for adherence to all four drug classes combined.

Multivariable Cox proportional hazards regression analysis was performed to evaluate the association between adherence and the composite endpoint of 2-year all-cause mortality and MAE. Associations are reported as hazard ratios (HR) with 95% confidence intervals (95% CI). Given the limited number of events, this model was specified as exploratory and included adherence to each of the four drug classes as separate covariates entered simultaneously. No additional clinical covariates were included to reduce the risk of overfitting; therefore, these results should be interpreted as hypothesis-generating and adjusted only for concomitant adherence status of the remaining GDMT pillars rather than as fully adjusted estimates. The composite endpoint was selected because no deaths occurred during follow-up among patients fully adherent to all four drug classes, which precluded reliable Cox regression modelling for all-cause mortality as an isolated endpoint. Prespecified subgroup analyses were conducted according to sex (male vs. female), age (<65 years vs. ≥65 years), and left ventricular ejection fraction (LVEF ≤ 30% vs. 31–40%), and body mass index (BMI < 30 kg/m^2^ vs. ≥30 kg/m^2^).

All statistical tests were two-sided, and a *p* value < 0.05 was considered statistically significant. Statistical analyses were performed using MedCalc Statistical Software, version 23.2.8, 2025 (MedCalc Software Ltd., Ostend, Belgium).

## 3. Results

Within the CaRD HF registry, 179 patients had newly diagnosed HFrEF during the study period. Three were not discharged on all four foundational GDMT classes, and 9 died before the first 6-month medication assessment; thus, 167 patients comprised the final analytic cohort (Figure A1). This HFrEF four-pillar subregistry was intentionally designed to examine the feasibility of maintaining quadruple GDMT after discharge in a real-world, non-randomized population encountered in daily practice (Table 1). The median available follow-up duration was 24 months. Median age was 64 years (IQR 55–71), 124 patients (74.3%) were men, median BMI was 28.1 kg/m^2^ (IQR 25.2–31.4), and median LVEF was 30% (IQR 25–35). The median N-terminal pro-B-type natriuretic peptide (NT-proBNP) concentration at presentation was 4793 pg/mL (IQR 1955–8501). The most frequent indication for index admission (Table A1) was HF (*n* = 87, 52.1%), followed by ST-elevation myocardial infarction (n = 39, 23.4%) and non-ST-elevation acute coronary syndrome (*n* = 17, 10.2%). Other admission reasons were planned evaluation (4.2%), outpatient follow-up (4.8%), coronary angiography (1.2%), device therapy (CRT/ICD/pacemaker; 1.2%), cerebrovascular insult (0.6%), pulmonary embolism (0.6%), ventricular tachycardia (0.6%) and other causes (1.2%). Medication questionnaires were completed by 167 patients at 6 months, 163 at 12 months and 152 at 24 months (Table A3, Table A4, Table A5 and Table A6).

### 3.1. Correlates of Adherence

At the 6-month assessment, 56/167 patients (33.5%) reported regular intake (≥80% of prescribed doses) of all four GDMT drug classes and were classified as adherent. The remaining 111 patients (66.5%) were classified as nonadherent, defined as irregular use or non-use of ≥1 GDMT class at 6 months. Compared with nonadherent patients, adherent patients were younger (median 61 vs. 66 years; *p* = 0.013), had higher BMI (median 29.4 vs. 27.7 kg/m^2^; *p* = 0.041), and were less likely to have arterial hypertension (51.8% vs. 73.0%; *p* = 0.006). In the overall cohort, arterial hypertension was present in 65.9%, dyslipidaemia in 65.9%, diabetes in 35.9%, coronary artery disease in 55.1%, atrial fibrillation in 26.9%, peripheral artery disease in 12.6%, chronic obstructive pulmonary disease or asthma in 10.2%, and prior stroke or transient ischemic attack in 9.0%; 49.7% reported nicotinism. Sex distribution, LVEF, New York Heart Association (NYHA) class and most other comorbidities and laboratory measures did not differ significantly between adherence groups (Table 1).

### 3.2. Socioeconomic Characteristics

Monthly household income distribution did not differ significantly between adherent and nonadherent patients (*p* = 0.115): <€330 (0.6% overall), €330–431 (14.4%), €432–832 (61.1%), and >€832 (24.0%). Education level was strongly associated with adherence (*p* < 0.001). In the adherent group, 23.2% had a bachelor’s/master’s degree versus 7.2% among nonadherent patients, whereas lack of elementary school education was less frequent in adherent patients (1.8% vs. 10.8%). Marital status (*p* = 0.975) and employment status (*p* = 0.191) were similar between groups (Table A2).

### 3.3. Medication Adherence over Time

**Figure 1:**
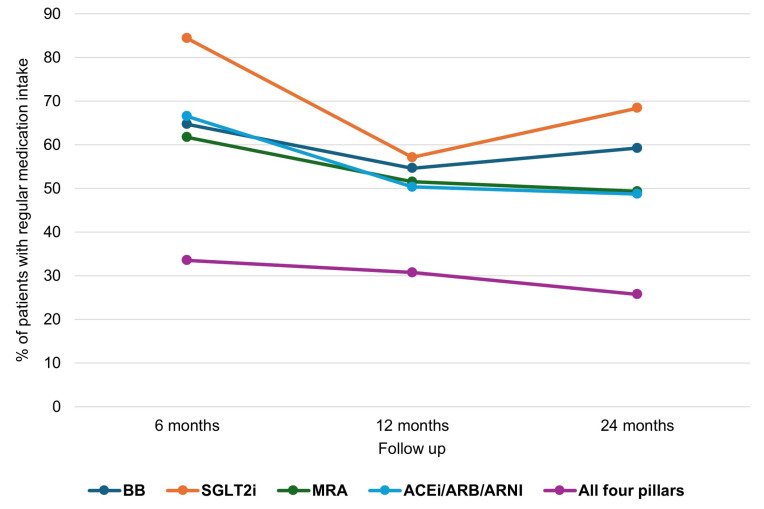
Adherence to heart failure medications over 6-, 12-, and 24-month follow-up. The x-axis denotes prespecified patient-level follow-up time after the index date, and the y-axis denotes the percentage (%) of patients reporting regular medication intake (≥80% of prescribed doses) for each GDMT class.

Regular adherence to each drug class at 6, 12 and 24 months is shown in Table A3, Table A4, Table A5 and Table A6 and Figure 1. At 24 months, regular use was reported by 59.2% for BBs, 48.7% for ACEi/ARB/ARNI, 49.3% for MRAs and 68.4% for SGLT2is; non-use (≤50% of prescribed doses) was reported by 21.1%, 20.4%, 21.1% and 17.8% of patients, respectively.

### 3.4. Beta-Blockers

**Figure A2:**
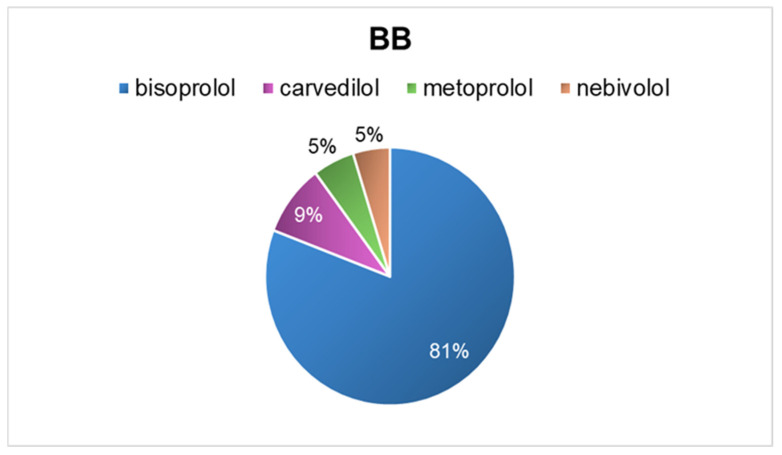
Distribution of BB agents at discharge.

At 6, 12 and 24 months, regular BB intake was reported by 64.7%, 54.6% and 59.2% of patients, respectively; 23.4%, 28.2% and 19.7% reported irregular use, and 12.0%, 17.2% and 21.1% reported non-use. Among patients not taking BBs regularly, the most frequently reported main reasons were forgetfulness/unintentional nonadherence (33.9%, 40.5% and 32.3% at 6, 12 and 24 months), belief that the medication was unnecessary (27.1%, 21.6% and 27.4%), poor tolerability/adverse effects (23.7%, 20.3% and 20.9%), and having not been instructed to take the medication (8.5%, 9.5% and 12.9%); discontinuation due to bradycardia was reported in 6.5–8.1%. Among BB users (regular or irregular), target doses were achieved in 25.2% at 6 months, 30.4% at 12 months and 33.3% at 24 months. In those not at target dose, the most common reported reasons were lack of attempted uptitration (52.7%, 44.7% and 43.4% at 6, 12 and 24 months) and hypotension or bradycardia (36.4%, 45.7% and 50.0%); fewer patients reported attempted but not maintained uptitration (5.3–8.2%) or dose reduction by a primary care physician (1.3–2.7%) (Table A3). The distribution of BB agents prescribed at hospital discharge is shown in Figure A2.

### 3.5. SGLT2 Inhibitors

**Figure A3:**
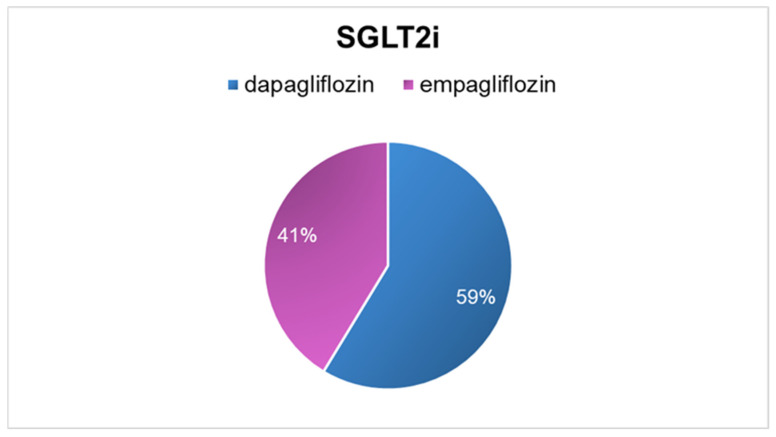
Distribution of SGLT2i agents at discharge.

Regular SGLT2i intake was reported by 84.4% at 6 months, 57.1% at 12 months and 68.4% at 24 months; irregular use by 6.6%, 15.3% and 13.8%; and non-use by 9.0%, 27.6% and 17.8%. Among patients not taking SGLT2i regularly, the most frequently reported reason at all time points was perceiving daily intake as unnecessary (53.8%, 52.9% and 52.1% at 6, 12 and 24 months), followed by not believing the medication was necessary (30.8%, 27.1% and 31.3%) and forgetfulness (15.4%, 20.0% and 16.6%) (Table A4). The distribution of SGLT2is prescribed at hospital discharge is presented in Figure A3.

### 3.6. Mineralocorticoid Receptor Antagonists

**Figure A4:**
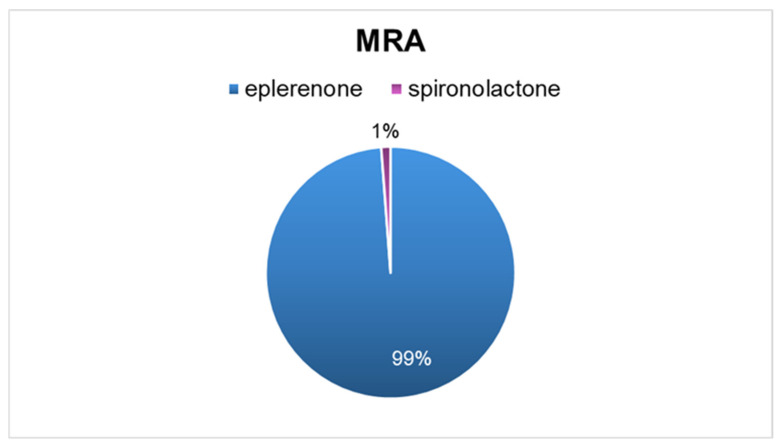
Distribution of MRA agents at discharge.

Regular MRA intake was reported by 61.7%, 51.5% and 49.3% at 6, 12 and 24 months; irregular use by 26.3%, 31.9% and 29.6%; and non-use by 12.0%, 16.6% and 21.1%. Among non-regular users, forgetfulness was the most frequently selected main reason (46.9%, 49.4% and 40.2% at 6, 12 and 24 months), followed by belief that the medication was unnecessary (21.9%, 29.1% and 29.9%). Not being instructed to take the medication was reported in 12.5%, 12.7% and 18.2%, while poor tolerability/adverse effects was reported in 8.8–18.7%. Among MRA users, target dosing was achieved in 72.8% at 6 months, 75.4% at 12 months and 77.5% at 24 months. In those not at target dose, reduced renal function or hyperkalaemia was the most common barrier (42.5%, 57.5% and 44.5% at 6, 12 and 24 months), alongside lack of attempted uptitration (42.5%, 30.3% and 22.2%). Hypotension accounted for 5.0% at 6 months, 6.1% at 12 months, and 22.2% at 24 months; attempted but not maintained uptitration accounted for 6.1–11.1% (Table A5). The distribution of MRAs prescribed at hospital discharge is presented in Figure A4.

### 3.7. ACEi/ARB/ARNI

**Figure A5:**
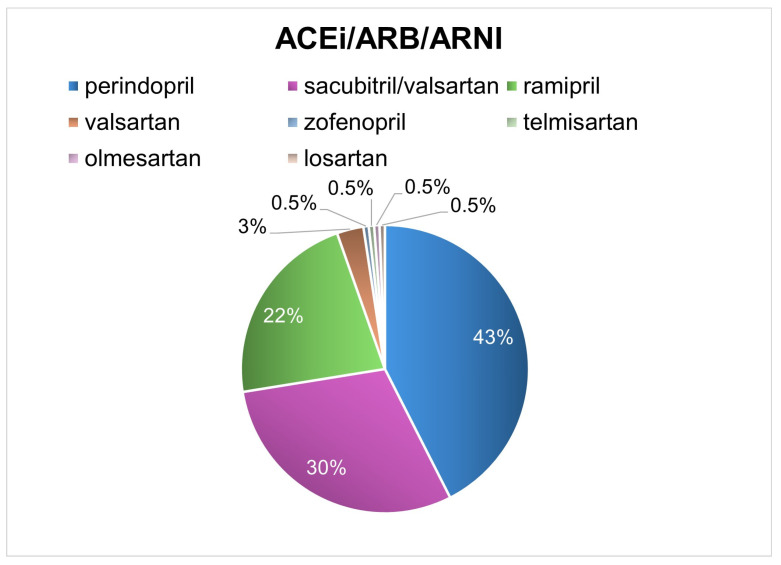
Distribution of ACEi/ARB/ARNI agents at discharge.

Regular ACEi/ARB/ARNI intake was reported by 66.5%, 50.3% and 48.7% at 6, 12 and 24 months; irregular use by 25.1%, 30.1% and 30.9%; and non-use by 8.4%, 19.6% and 20.4%. Among non-regular users, poor tolerability/adverse effects was the leading main reason (42.8%, 45.7% and 48.7% at 6, 12 and 24 months), followed by forgetfulness (39.3%, 28.4% and 23.1%) and belief that the medication was unnecessary (12.5%, 22.2% and 24.4%). Not being instructed to take the medication was reported by 3.7–5.4%. Among ACEi/ARB/ARNI users, target doses were achieved in 41.8% at 6 months, 44.3% at 12 months and 49.6% at 24 months. In patients not at target dose, hypotension was the most frequently reported barrier (50.5%, 65.8% and 65.6% at 6, 12 and 24 months). Hyperkalaemia accounted for 9.6–14.7%, lack of attempted uptitration for 31.5% at 6 months (falling to 12.3% at 12 months and 6.6% at 24 months), and attempted but not maintained uptitration for 7.9–13.1% (Table A6). The distribution of ACEi/ARB/ARNI prescribed at hospital discharge is shown in Figure A5.

### 3.8. Clinical Outcomes

**Figure 2:**
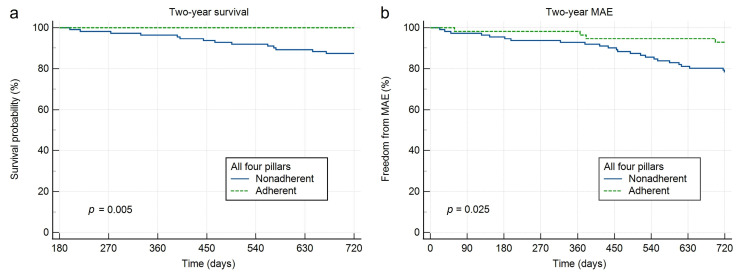
Kaplan–Meier curves for 2-year survival and freedom from major adverse events according to GDMT adherence. Kaplan–Meier curves compare patients adherent to all four GDMT pillars within the first 6 months of follow-up with nonadherent patients (defined as nonadherence to at least one GDMT class). Panel (**a**) shows 2-year all-cause survival, and panel (**b**) shows freedom from major adverse events (MAE).

**Figure A6:**
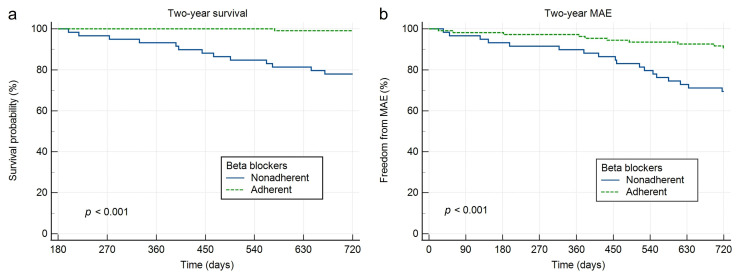
Kaplan–Meier curves illustrate 2-year all-cause survival (**a**) and freedom from major adverse events (**b**) stratified by adherence to BB.

**Figure A7:**
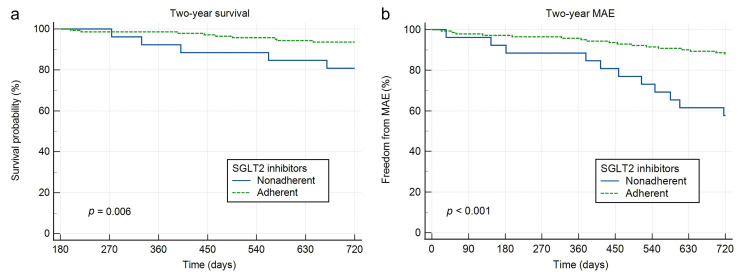
Kaplan–Meier curves illustrate 2-year all-cause survival (**a**) and freedom from major adverse events (**b**) stratified by adherence to SGLT2i.

**Figure A8:**
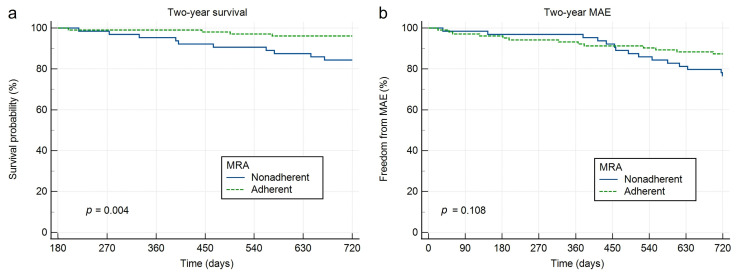
Kaplan–Meier curves show 2-year all-cause survival (**a**) and freedom from major adverse events (**b**) stratified by adherence to MRA.

**Figure A9:**
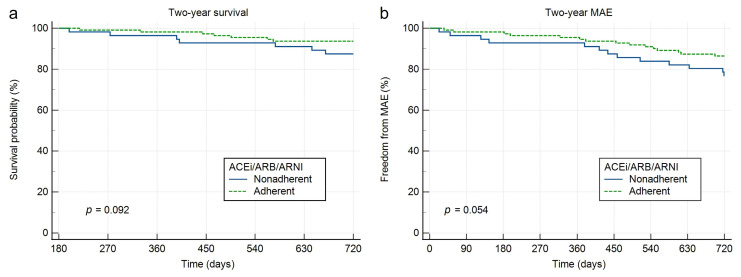
Kaplan–Meier curves depict 2-year all-cause survival (**a**) and freedom from major adverse events (**b**) according to adherence to ACEi/ARB/ARNI.

Over 24 months, 15 patients died (all-cause mortality 9.0%) and 31 experienced a MAE (18.6%). The most common MAE component was HF decompensation requiring hospitalization (12.6%), followed by dialysis or worsening renal function (2.4%); acute coronary syndrome, sepsis, heart transplantation, cerebrovascular insult and thromboembolic/peripheral vascular events each occurred in ≤1.2%. In exploratory comparisons by adherence status defined at 6 months, mortality (0.0% vs. 13.5%, *p* = 0.004) and MAE (8.9% vs. 23.4%, *p* = 0.023) were lower in the adherent group (Table 2). Kaplan–Meier curves for 2-year all-cause mortality and MAE stratified by adherence to all four GDMT pillars are shown in Figure 2, while the corresponding analyses for individual GDMT drug classes are presented in Figure A6, Figure A7, Figure A8 and Figure A9.

**Figure 3:**
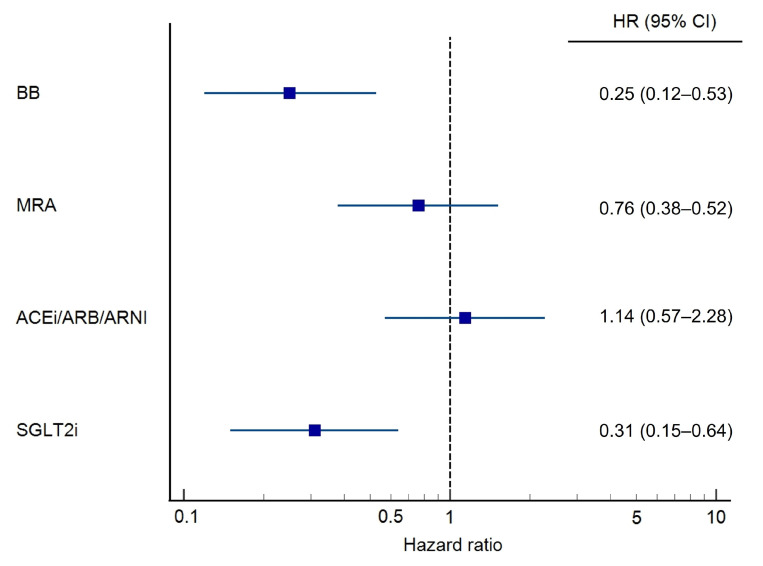
Multivariable Cox regression analysis of adherence to individual GDMT drug classes and the composite endpoint.

Figure 3 presents the results of the multivariable regression analysis, demonstrating that adherence to BB (HR 0.25, 95% CI 0.12–0.53, *p* < 0.001) and SGLT2i (HR 0.31, 95% CI 0.15–0.64, *p* = 0.002) was significantly associated with a lower risk of mortality or MAE, whereas adherence to ACEi/ARB/ARNI and MRA did not reach statistical significance. Table A7 shows prespecified subgroup analyses stratified by sex, age, LVEF, and BMI.

## 4. Discussion

Our findings further highlight the critical distinction between early initiation and durable implementation of GDMT in HFrEF. While contemporary guidelines and sequencing frameworks have successfully shifted practice towards rapid or simultaneous initiation of the four foundational drug classes [1,2,11,12,13], our data demonstrate that initiation alone does not ensure sustained adherence or dose optimisation over time. Importantly, this predefined CaRD subregistry was conceived to examine whether such an intensive strategy can be implemented and maintained outside randomized trials, in patients seen in routine practice once they leave hospital on all four pillars. Even within this selected cohort, only approximately 25% of 24-month survivors reported regular intake of all four GDMT pillars, highlighting a substantial gap between guideline recommendations and durable real-world implementation.

Importantly, differences in adherence were not explained by markers of HF severity. Adherent and nonadherent patients did not differ significantly LVEF, NYHA class, or NT-proBNP levels, suggesting that nonadherence cannot be attributed solely to more advanced disease or haemodynamic instability. This observation aligns with registry data showing that behavioural, cognitive and healthcare-system factors often outweigh clinical severity as determinants of long-term GDMT persistence [15,16,17,18,19,21].

Drug-class-specific analyses revealed distinct and clinically relevant barriers. For BBs, lack of attempted dose up-titration was the dominant reason for failure to achieve target doses, accounting for approximately 40–50% of missed targets across follow-up. This finding is consistent with prior reports identifying therapeutic inertia as a major obstacle to BB optimisation in HFrEF [18,19]. Given the strong evidence base for BBs in reducing mortality and HF hospitalizations [6,7,8], our results emphasise the need for protocol-driven titration pathways and structured follow-up beyond the early post-discharge period.

For ACEi/ARB/ARNI therapy, poor tolerability and hypotension were the most frequently reported reasons for both non-regular use and failure to reach target doses. These findings reflect real-world haemodynamic constraints and are in line with previous studies reporting hypotension as a leading limitation to renin-angiotensin system inhibitor optimization [14,18]. Notably, an increasing proportion of patients also reported low perceived necessity of therapy over time, suggesting that symptomatic improvement may undermine long-term persistence unless prognostic benefits are repeatedly reinforced [22,23].

SGLT2i showed the highest overall persistence but a pronounced decline at mid-term follow-up, driven predominantly by perceptions that daily intake was unnecessary. Large randomised trials have demonstrated prognostic benefits of SGLT2i in HFrEF irrespective of diabetes status [9,10], yet our findings suggest that this paradigm shift is not fully translated into patient understanding. Structured education explicitly addressing indications and expected benefits may therefore be essential to sustain long-term adherence.

MRAs exhibited relatively high target-dose achievement among users, but regular intake declined steadily over time. While renal dysfunction and hyperkalaemia remained key barriers to dose optimisation, unintentional nonadherence and unclear instructions accounted for a substantial proportion of discontinuation. Similar challenges have been reported in real-world cohorts, highlighting the importance of systematic laboratory monitoring and clear communication to maintain MRA therapy safely and effectively [4,5,15,16,17].

Socioeconomic analyses further contextualise these findings. Education level, but not income, employment status, or marital status, was strongly associated with adherence, supporting the concept that health literacy rather than socioeconomic position per se is a key determinant of GDMT persistence. This pattern is consistent with broader adherence literature, which identifies understanding of disease and treatment as central drivers of long-term medication use [21]. Tailored educational interventions, simplified regimens and repeated medication reviews may therefore be particularly beneficial in patients with lower educational attainment.

Although exploratory and subject to important limitations, the observed associations between adherence to all four GDMT classes and lower mortality and MAE are directionally consistent with registry data linking higher guideline adherence and more complete dose optimization to improved outcomes [18,19]. These comparisons should be interpreted cautiously given potential confounding and survivorship bias, as adherence was defined at 6 months. In the exploratory Cox model, adherence to BB and SGLT2i retained statistical significance, whereas adherence to ACEi/ARB/ARNI and MRA did not. This should not be interpreted as evidence that the latter therapies are less important. Because adherence to the four pillars is clinically interrelated, simultaneous entry of all four adherence variables introduces correlation and potential collinearity, while the limited number of events reduces statistical power. Under these conditions, non-significance likely reflects limited precision, exposure correlation, and residual confounding rather than absence of benefit.

Our findings should also be interpreted in the context of worsening HF and the vulnerable phase after diagnosis or decompensation, where long-term prognosis depends not only on early initiation of foundational therapies but also on their sustained continuation and optimisation over time. Recent reviews have emphasised that contemporary HF management requires ongoing reassessment of clinical stability, patient education, and reinforcement of the prognostic value of treatment beyond early symptom improvement [23].

Taken together, our findings suggest that the next major challenge in HFrEF management is no longer whether quadruple GDMT can be initiated early, but how it can be maintained, titrated and integrated into long-term care pathways. Multidisciplinary follow-up models, protocolised titration strategies and repeated patient-centred education-similar in principle to high-intensity optimisation programmes such as STRONG-HF, may be required to close the persistent gap between evidence-based recommendations and real-world outcomes [20].

### Limitations

This study has several important limitations. First, it was conducted at a single tertiary centre and included a moderate sample size, which may limit external validity. Second, the analytic cohort was intentionally restricted to patients with newly diagnosed HFrEF who were discharged on all four foundational GDMT classes and survived to the first 6-month medication assessment. The study therefore represents a selected subgroup of quadruple-eligible early survivors, enriched for clinically stable patients able to tolerate early comprehensive therapy, and cannot be generalised to the entire population of newly diagnosed HFrEF patients. Third, this analysis represents a predefined subregistry within a broader HF registry and reflecting routine introduction or optimisation of SGLT2i therapy; accordingly, the present findings should not be extrapolated to the full registry population. Fourth, the Cox analyses were exploratory and limited by the low event rate (15 deaths and 31 MAEs). The primary multivariable model incorporated adherence variables only and was not adjusted for age, LVEF, NT-proBNP, BMI, comorbidities, or other baseline characteristics. Instead, stratified subgroup analyses were conducted across these four prespecified variables. Given the limited number of events, residual confounding, model instability, and limited statistical power should be considered when interpreting the results. Fifth, we did not perform a formal baseline comparison between included and excluded patients, and the excluded subgroup was small and clinically heterogeneous. Ethnicity was not systematically recorded in the registry; given the single-centre Croatian setting the cohort is likely relatively homogeneous, but this variable could not be analysed and should be regarded as a limitation. Sixth, adherence was assessed using a structured study questionnaire that was not formally validated against pharmacy refill data, pill counts, or electronic monitoring. Self-report may overestimate adherence, the ≥80% threshold is pragmatic, and dose timing or refill gaps were not captured. Although electronic medical record review provided supportive information on treatment activation or continuation in primary care, neither self-report nor record review could fully confirm actual medication ingestion or perfectly distinguish primary nonadherence from later discontinuation. Seventh, the questionnaire-based approach could not fully distinguish patient nonadherence from clinician-directed discontinuation or dose reduction due to intolerance or clinical decision-making. Reasons related to tolerability or clinical instability were recorded descriptively, but early deaths were excluded because adherence status could not be classified, and their relationship to treatment intolerance could not be reliably determined from the available registry data. In addition, not all potential adherence barriers were systematically recorded; in particular, cost burden and regimen complexity were not prospectively captured. Eighth, because adherence status was defined at 6 months, outcome comparisons are susceptible to survivorship and immortal-time bias and should be interpreted as hypothesis-generating. We did not perform landmark or time-dependent analyses. Finally, MAE components were not independently adjudicated and the composite included heterogeneous events; component analyses are descriptive.

## 5. Conclusions

In this real-world, non-randomized subregistry of patients with newly diagnosed HFrEF who were discharged on quadruple GDMT and survived to early follow-up, early initiation during hospitalisation was feasible, but long-term adherence and target-dose attainment remained suboptimal. These findings apply primarily to a selected four-pillar cohort encountered in everyday clinical practice rather than to the full population of newly diagnosed HFrEF. Structured follow-up, repeated patient education, and protocolised titration pathways may be required to sustain quadruple therapy and optimise dosing over time.

## Figures and Tables

**Table 1:** Baseline characteristic of study population.

Variables	Total (n = 167)	Adherent (n = 56)	Nonadherent (n = 111)	*p*-Value
Age (years)	64 (55–71)	61 (52–68)	66 (58–72)	0.013 *
Sex (%)				0.200
Male	124 (74.3)	45 (80.4)	79 (71.2)	
Female	43 (25.7)	11 (19.6)	32 (28.8)	
BMI (kg/m^2^)	28.1 (25.2–31.4)	29.4 (25.9–34.2)	27.7 (24.9–30.9)	0.041 *
Co-morbidities (%)				
Arterial hypertension	110 (65.9)	29 (51.8)	81 (73.0)	0.006 *
Dyslipidemia	110 (65.9)	38 (67.9)	72 (64.9)	0.700
Diabetes	60 (35.9)	19 (33.9)	41 (36.9)	0.702
Prediabetes	63 (37.7)	23 (41.1)	40 (36.0)	0.526
CAD	92 (55.1)	32 (57.1)	60 (54.1)	0.705
PAD	21 (12.6)	6 (10.7)	15 (13.5)	0.607
Atrial fibrillation	45 (26.9)	16 (28.6)	29 (26.1)	0.737
Prior stroke or TIA	15 (9.0)	7 (12.5)	8 (7.2)	0.259
COPD or asthma	17 (10.2)	8 (14.3)	9 (8.1)	0.213
Nicotinismus	83 (49.7)	29 (51.8)	54 (48.6)	0.702
Clinical characteristics				
HR (b.p.m.)	96 ± 21	95 ± 20	96 ± 21	0.816
SBP (mmHg)	136 ± 24	134 ± 22	137 ± 25	0.436
DBP (mmHg)	86 ± 15	85 ± 15	87 ± 15	0.357
LVEF (%)	30 (25–35)	35 (27–35)	30 (25–35)	0.444
NYHA classification				0.112
I	8 (4.8)	4 (7.1)	4 (3.6)	
II	65 (38.9)	27 (48.2)	38 (34.2)	
III	80 (47.9)	23 (41.1)	57 (51.4)	
IV	14 (8.4)	2 (3.6)	12 (10.8)	
Biological data				
Hemoglobin (g/L)	139 (130–150)	141 (132–151)	138 (127–150)	0.257
Hematocrit (L/L)	0.42 ± 0.05	0.43 ± 0.04	0.42 ± 0.05	0.300
MCV (fL)	90.2 (86.4–93.6)	90.2 (86.0–93.1)	90.2 (86.5–93.8)	0.776
RCDW (%)	14.0 (13.3–14.8)	14.0 (13.3–14.8)	14.0 (13.4–14.8)	0.710
Glucose (mmol/L)	6.8 (5.6–8.9)	6.6 (5.7–8.2)	6.9 (5.4–9.2)	0.475
HbA1c (%)	6.0 (5.7–6.8)	6.0 (5.7–6.7)	6.1 (5.7–6.8)	0.787
Creatinine (µmol/L)	85 (73–108)	88 (73–109)	85 (73–106)	0.655
eGFR (mL/min/1.73 m^2^)	77 (59–93)	80 (59–94)	75 (59–93)	0.797
Urea (mmol/L)	6.9 (5.3–9.3)	6.7 (5.2–9.4)	7.0 (5.3–8.9)	0.856
Total cholesterol (mmol/L)	4.9 (3.8–6.0)	5.2 (4.1–6.1)	4.5 (3.6–5.8)	0.060
LDL-C (mmol/L)	3.0 (2.2–3.9)	3.6 (2.6–4.3)	2.9 (2.0–3.9)	0.057
HDL-C (mmol/L)	1.2 (1.0–1.5)	1.3 (1.0–1.6)	1.2 (1.0–1.3)	0.092
Triglycerides (mmol/L)	1.1 (0.9–1.5)	1.1 (0.9–1.7)	1.1 (0.9–1.5)	0.641
CRP (mg/L)	7.8 (3.6–18.8)	7.6 (4.1–25.0)	8.4 (3.5–17.4)	0.725
Potassium (mmol/L)	4.2 (4.0–4.6)	4.3 (4.0–4.5)	4.2 (4.0–4.6)	0.358
Sodium (mmol/L)	139 (137–141)	139 (137–141)	139 (137–140)	0.715
Albumin (g/L)	39 ± 4	39 ± 4	39 ± 5	0.277
NT-proBNP (pg/mL)	4793 (1955–8501)	3373 (1853–7939)	5135 (2032–7939)	0.598

BMI: body mass index; CAD: coronary artery disease; COPD: chronic obstructive pulmonary disease; CRP: C-reactive protein; DBP: diastolic blood pressure; eGFR: estimated glomerular filtration rate; HbA1c: hemoglobin A1c; HDL-C: high-density lipoprotein cholesterol; HR: heart rate; LDL-C: low-density lipoprotein cholesterol; LVEF: left ventricular ejection fraction; NT-proBNP: N-terminal pro-B-type natriuretic peptide; NYHA: New York Heart Association; PAD: peripheral artery disease; RCDW: red cell distribution width; SBP: systolic blood pressure; TIA: transient ischemic attack. Missing data: albumin (38), CRP (3), creatinine (1), HbA1c (6), HDL-C (2), LDL-C (2), NT-proBNP (3), potassium (1), sodium (2), total cholesterol (2), triglycerides (3), urea (1). *: *p* < 0.05.

**Table 2:** Baseline characteristic of study population.

Variables	Total (n = 167)	Adherent (n = 56)	Nonadherent (n = 111)	*p*-Value
All-cause mortality (%)	15 (9.0)	0 (0.0)	15 (13.5)	0.004 *
Major adverse events (%)	31 (18.6)	5 (8.9)	26 (23.4)	0.023 *
Heart failure decompensation	21 (12.6)	2 (3.6)	19 (17.1)	
Acute coronary syndrome	2 (1.2)	2 (3.6)	0 (0.0)	
Cerebrovascular insult	1 (0.6)	0 (0.0)	1 (0.9)	
Sepsis	1 (0.6)	1 (1.8)	0 (0.0)	
Dialysis or worsening renal function	4 (2.4)	0 (0.0)	4 (3.6)	
Heart transplantation	1 (0.6)	0 (0.0)	1 (0.9)	
Thromboembolic or peripheral vascular event	1 (0.6)	0 (0.0)	1 (0.9)	

*: *p* < 0.05.

## Data Availability

The data underlying this article cannot be shared publicly due to the privacy of the individuals who participated in the study. The data will be shared upon reasonable request made to the corresponding author.

## Supplementary Materials

The following supporting information can be downloaded at: https://www.mdpi.com/article/10.3390/jcm15062127/s1, As this is an observational study based on registry data, the STROBE checklist [24] is provided in the Supplementary Material.

## Abbreviations

The following abbreviations are used in this manuscript:

ACEi	Angiotensin-converting enzyme inhibitor
ARB	Angiotensin receptor blocker
ARNI	Angiotensin receptor–neprilysin inhibitor
BB	Beta-blocker
BMI	Body mass index
CI	Confidence interval
CRT	Cardiac resynchronization therapy
ESC	European Society of Cardiology
GDMT	Guideline-directed medical therapy
HFrEF	Heart failure with reduced ejection fraction
HR	Hazard ratio
ICD	Implantable cardioverter-defibrillator
IQR	Interquartile range
LVEF	Left ventricular ejection fraction
MEA	Major adverse event
MRA	Mineralocorticoid receptor antagonist
NT-proBNP	N-terminal pro-B-type natriuretic peptide
NYHA	New York Heart Association
SGLT2i	Sodium–glucose cotransporter-2 inhibitor

## Appendix A

**Table A1:** Indications for index hospital admission among patients with newly diagnosed HFrEF.

Indication	N	%
Heart failure	87	52.1
ST-elevation myocardial infarction	39	23.4
Non-ST-elevation acute coronary syndrome	17	10.2
Planned evaluation	7	4.2
Outpatient follow-up	8	4.8
Coronary angiography	2	1.2
CRT/ICD/pacemaker	2	1.2
Cerebrovascular insult	1	0.6
Pulmonary embolism	1	0.6
Ventricular tachycardia	1	0.6
Other	2	1.2

CRT: cardiac resynchronization therapy; ICD: implantable cardioverter-defibrillator.

**Table A2:** Socioeconomic characteristics of the study population.

Variables	Total (n = 167)	Adherent (n = 56)	Nonadherent (n = 111)	*p*-Value
Monthly household income (%)				0.115
<330 €	1 (0.6)	0 (0.0)	1 (0.9)	
330–431 €	24 (14.4)	4 (7.1)	20 (18.0)	
432–832 €	102 (61.1)	34 (60.7)	68 (61.3)	
>832 €	40 (24.0)	18 (32.1)	22 (19.8)	
Education level (%)				<0.001 *
No elementary school	13 (7.8)	1 (1.8)	12 (10.8)	
Elementary school graduate	58 (34.7)	12 (21.4)	46 (41.4)	
High school graduate	75 (44.9)	30 (53.6)	45 (40.5)	
Bachelor’s/master’s degree	21 (12.6)	13 (23.2)	8 (7.2)	
Marital status (%)				0.975
Married	117 (70.1)	40 (71.4)	77 (69.4)	
Single	19 (11.4)	6 (10.7)	13 (11.7)	
Divorced	17 (10.2)	5 (8.9)	12 (10.8)	
Widow/widower	14 (8.4)	5 (8.9)	9 (8.1)	
Employment status (%)				0.191
Employed	54 (32.3)	24 (42.9)	30 (27.0)	
Unemployed	20 (12.0)	7 (12.5)	13 (11.7)	
Retired	89 (53.3)	24 (42.9)	65 (58.6)	
Occasionally employed	4 (2.4)	1 (1.8)	3 (2.7)	

*: *p* < 0.05.

**Table A3:** Adherence-related referral implementation for beta-blocker therapy at 6-, 12-, and 24-month follow-up.

Questions	6 Months	12 Months	24 Months
Do you take a beta blocker?	n = 167	n = 163	n = 152
Regularly (>80% of the time)	108 (64.7%)	89 (54.6%)	90 (59.2%)
Irregularly (50–80% of the time)	39 (23.4%)	46 (28.2%)	30 (19.7%)
Not taking (<50% of the time)	20 (12.0%)	28 (17.2%)	32 (21.1%)
If not taking a beta blocker regularly (n = irregular or not taking): what is the main reason?	n = 59	n = 74	n = 62
Poor tolerability/adverse effects	14 (23.7%)	15 (20.3%)	13 (20.9%)
Was not instructed to take the medication	5 (8.5%)	7 (9.5%)	8 (12.9%)
Does not believe the medication is necessary	16 (27.1%)	16 (21.6%)	17 (27.4%)
Forgetfulness/non-intentional non-adherence	20 (33.9%)	30 (40.5%)	20 (32.3%)
Discontinued due to bradycardia	4 (6.8%)	6 (8.1%)	4 (6.5%)
Are you receiving the target dose of a beta blocker? (n = regular or irregular users)	n = 147	n = 135	n = 120
Yes	37 (25.2%)	41 (30.4%)	44 (33.3%)
If not receiving the target dose of a beta blocker (n = regular or irregular users not on target dose): what is the main reason?	n = 110	n = 94	n = 76
Hypotension or bradycardia	40 (36.4%)	43 (45.7%)	38 (50.0%)
Dose up-titration was not attempted	58 (52.7%)	42 (44.7%)	33 (43.4%)
Dose up-titration was attempted but not maintained	9 (8.2%)	7 (7.5%)	4 (5.3%)
Dose was reduced by the primary care physician	3 (2.7%)	2 (2.1%)	1 (1.3%)

**Table A4:** Adherence-related referral implementation for SGLT2 inhibitor therapy at 6-, 12-, and 24-month follow-up.

Questions	6 Months	12 Months	24 Months
Do you take a SGLT2 inhibitor?	n = 167	n = 163	n = 152
Regularly (>80% of the time)	141 (84.4%)	93 (57.1%)	104 (68.4%)
Irregularly (50–80% of the time)	11 (6.6%)	25 (15.3%)	21 (13.8%)
Not taking (<50% of the time)	15 (9.0%)	45 (27.6%)	27 (17.8%)
If not taking a SGLT2 inhibitor regularly (n = irregular or not taking): what is the main reason?	n = 26	n = 70	n = 48
Perceives that daily intake of the medication is unnecessary	14 (53.8%)	37 (52.9%)	25 (52.1%)
Does not believe the medication is necessary	8 (30.8%)	19 (27.1%)	15 (31.3%)
Forgetfulness/non-intentional non-adherence	5 (15.4%)	14 (20.0%)	8 (16.6%)

**Table A5:** Adherence-related referral implementation for mineralocorticoid receptor antagonist therapy at 6-, 12-, and 24-month follow-up.

Questions	6 Months	12 Months	24 Months
Do you take a MRA?	n = 167	n = 163	n = 152
Regularly (>80% of the time)	103 (61.7%)	84 (51.5%)	75 (49.3%)
Irregularly (50–80% of the time)	44 (26.3%)	52 (31.9%)	45 (29.6%)
Not taking (<50% of the time)	20 (12.0%)	27 (16.6%)	32 (21.1%)
If not taking a MRA regularly (n = irregular or not taking): what is the main reason?	n = 64	n = 79	n = 77
Poor tolerability/adverse effects	12 (18.7%)	7 (8.8%)	9 (11.7%)
Was not instructed to take the medication	8 (12.5%)	10 (12.7%)	14 (18.2%)
Does not believe the medication is necessary	14 (21.9%)	23 (29.1%)	23 (29.9%)
Forgetfulness/non-intentional non-adherence	30 (46.9%)	39 (49.4%)	31 (40.2%)
Are you receiving the target dose of a MRA? (n = regular or irregular users)	n = 147	n = 134	n = 120
Yes	107 (72.8%)	101 (75.4%)	93 (77.5%)
If not receiving the target dose of a MRA (n = regular or irregular users not on target dose): what is the main reason?	n = 40	n = 33	n = 27
Reduced renal function or hyperkalaemia	17 (42.5%)	19 (57.5%)	12 (44.5%)
Hypotension	2 (5.0%)	2 (6.1%)	6 (22.2%)
Dose up-titration was not attempted	17 (42.5)	10 (30.3%)	6 (22.2%)
Dose up-titration was attempted but not maintained	4 (10.0%)	2 (6.1%)	3 (11.1%)

**Table A6:** Adherence-related referral implementation for ACEi/ARB/ARNI therapy at 6-, 12-, and 24-month follow-up.

Questions	6 Months	12 Months	24 Months
Do you take a ACEi/ARB/ARNI?	n = 167	n = 163	n = 152
Regularly (>80% of the time)	111 (66.5%)	82 (50.3%)	74 (48.7%)
Irregularly (50–80% of the time)	42 (25.1%)	49 (30.1%)	47 (30.9%)
Not taking (<50% of the time)	14 (8.4%)	32 (19.6%)	31 (20.4%)
If not taking a ACEi/ARB/ARNI regularly (n = irregular or not taking): what is the main reason?	n = 56	n = 81	n = 78
Poor tolerability/adverse effects	24 (42.8%)	37 (45.7%)	38 (48.7%)
Was not instructed to take the medication	3 (5.4%)	3 (3.7%)	3 (3.8%)
Does not believe the medication is necessary	7 (12.5%)	18 (22.2%)	19 (24.4%)
Forgetfulness/non-intentional non-adherence	22 (39.3%)	23 (28.4%)	18 (23.1%)
Are you receiving the target dose of a ACEi/ARB/ARNI? (n = regular or irregular users)	n = 153	n = 131	n = 121
Yes	64 (41.8%)	58 (44.3%)	60 (49.6%)
If not receiving the target dose of a ACEi/ARB/ARNI (n = regular or irregular users not on target dose): what is the main reason?	n = 89	n = 73	n = 61
Hyperkalaemia	9 (10.1%)	7 (9.6%)	9 (14.7%)
Hypotension	45 (50.5%)	48 (65.8%)	40 (65.6%)
Dose up-titration was not attempted	28 (31.5%)	9 (12.3%)	4 (6.6%)
Dose up-titration was attempted but not maintained	7 (7.9%)	9 (12.3%)	8 (13.1%)

**Table A7:** Subgroup Cox regression analysis of adherence to guideline-directed medical therapy and the composite outcome stratified by age, sex, LVEF, and BMI.

	HR	95% CI	*p*-Value
Male			
Beta-blockers	0.29	0.12–0.70	0.006 *
SGLT2 inhibitors	0.25	0.10–0.60	0.002 *
MRAs	0.88	0.38–2.06	0.774
ACEi/ARB/ARNI	1.34	0.55–3.27	0.514
Female			
Beta-blockers	0.11	0.02–0.59	0.010 *
SGLT2 inhibitors	0.63	0.16–2.57	0.520
MRAs	0.43	0.11–1.56	0.199
ACEi/ARB/ARNI	1.16	0.32–4.20	0.820
<65 years			
Beta-blockers	0.25	0.07–0.87	0.029 *
SGLT2 inhibitors	0.30	0.08–1.05	0.060
MRAs	0.93	0.25–3.48	0.915
ACEi/ARB/ARNI	1.02	0.32–3.26	0.971
≥65 years			
Beta-blockers	0.28	0.10–0.74	0.011 *
SGLT2 inhibitors	0.27	0.11–0.69	0.006 *
MRAs	0.63	0.27–1.45	0.278
ACEi/ARB/ARNI	1.41	0.58–3.44	0.452
LVEF ≤ 30%			
Beta-blockers	0.31	0.12–0.84	0.021 *
SGLT2 inhibitors	0.29	0.11–0.76	0.012 *
MRAs	0.72	0.26–1.96	0.516
ACEi/ARB/ARNI	1.11	0.41–2.97	0.843
LVEF 31–40%			
Beta-blockers	0.17	0.06–0.60	0.005 *
SGLT2 inhibitors	0.35	0.11–1.08	0.069
MRAs	0.90	0.31–2.59	0.847
ACEi/ARB/ARNI	1.22	0.42–3.58	0.719
BMI < 30 kg/m^2^			
Beta-blockers	0.22	0.09–0.53	0.001 *
SGLT2 inhibitors	0.40	0.17–1.86	0.040 *
MRAs	0.89	0.38–2.08	0.787
ACEi/ARB/ARNI	0.85	0.39–1.86	0.680
BMI ≥ 30 kg/m^2^			
Beta-blockers	0.47	0.09–2.59	0.389
SGLT2 inhibitors	0.05	0.01–0.50	0.011 *
MRAs	0.51	0.13–1.98	0.327
ACEi/ARB/ARNI	5.09	0.53–48.44	0.157

*: *p* < 0.05.
